# Carbon-Based Multifunctional Nanomaterials

**DOI:** 10.3390/nano14191600

**Published:** 2024-10-03

**Authors:** Huitao Yu, Wei Feng

**Affiliations:** Tianjin Key Laboratory of Composite and Functional Materials, School of Materials Science and Engineering, Tianjin University, Tianjin 300350, China; huitaoy@tju.edu.cn

## 1. Introduction

Carbon-based nanomaterials have garnered widespread attention and application because of their exceptional electrical conductivity, thermal conductivity, mechanical strength, and optical properties. Common representative materials mainly include graphene, carbon nanotubes, fullerenes, and carbon quantum dots [1,2,3]. These materials not only attract significant interest because of their unique physical and chemical properties but also because they demonstrate immense potential in a wide range of fields. Therefore, exploring the key characteristics, application domains, and development prospects of carbon-based multifunctional nanomaterials is crucial for gaining a comprehensive understanding of this field and advancing its progress.

The exceptional properties of carbon-based multifunctional nanomaterials make them highly prominent in the fields of electronic devices and energy storage. Carbon materials can be classified into two-dimensional, one-dimensional, and zero-dimensional categories. For instance, two-dimensional graphene exhibits remarkable electrical and thermal conductivity, granting it significant potential in high-performance electronic devices, sensors, and supercapacitors. One-dimensional carbon nanotubes, with their unique structure, demonstrate outstanding mechanical strength and electrical conductivity, making them ideal for enhancing composite materials and fabricating nanoscale electronic devices [4]. Zero-dimensional materials such as fullerenes and carbon quantum dots, owing to their high specific surface area and excellent optical properties, excel in areas such as catalysis, optoelectronic devices, and bioimaging [5]. Moreover, carbon-based multifunctional nanomaterials show great promise in environmental protection and biomedical applications. For example, graphene can be employed for detecting pollutants in air and water. Carbon quantum dots, because of their excellent biocompatibility and strong fluorescence, are emerging as a research focus for the next generation of biomarkers and imaging probes [6]. Fullerenes and their derivatives also hold significant potential in drug delivery and antioxidant therapy.

Despite the remarkable performance advantages of carbon-based multifunctional nanomaterials in various fields, there are still some challenges in their practical application. First, the synthesis and functionalization processes of these nanomaterials need to be further optimized to reduce costs and improve consistency and reproducibility [7,8,9]. For example, structural defects and irregularities in the production of graphene and carbon nanotubes can affect the stability and reliability of their performance [10]. To address these challenges, researchers are exploring novel synthesis methods and functionalization techniques to meet the needs of various applications. Furthermore, the integration of nanomaterials with other functional materials could further improve their properties. In summary, carbon-based multifunctional nanomaterials, as a new generation of high-performance materials, are at the forefront of materials science research because of their exceptional physical and chemical properties and their broad application potential.

## 2. An Overview of Published Articles

Christoph et al. (Contribution 1) present a renewable source of carbon nanoparticles. They report the synthesis and material and biological characterization of two colloidal suspensions of CQDs in water derived from lignin-based carbon. The material exhibits non-toxic and biocompatible properties, laying a new foundation for the use of lignin-derived CQDs in tissue engineering applications.

Ioni et al. (Contribution 2) demonstrate a method for preparing a tailored composite based on nickel nanoparticles on the reduced graphene oxide surface using supercritical isopropanol treatment. The reduction in Ni salts enables the deoxygenation of graphene, with nickel nanoparticles uniformly distributed on the surface of graphene, and the composite exhibits magnetic properties. This enhances the understanding of the mechanism of composite fabrication in supercritical isopropanol.

Li et al. (Contribution 3) modified graphene with dopamine (PDA) at the interface, and then constructed multilayer composite films with boron nitride and bacterial cellulose. PDA improved the interface compatibility between the hybrid fillers and the matrix, increasing the material density and stabilizing the thermal conduction pathways. The composite film exhibited significant improvements in thermal conductivity and tensile strength.

Oswald et al. (Contribution 4) investigate the effects of thermally evaporated C60 (*n*-type) and Pentacene (*p*-type) thin films on the in-plane charge transport properties of large-area CVD graphene under vacuum. C60 induces a decrease in Fermi energy and an increase in Fermi energy in graphene. The increase in charge carriers was accompanied by a reduction in charge mobility. Interestingly, the contact resistance was not significantly affected by the deposition of the organic molecules.

Sun et al. (Contribution 5) provide a comprehensive overview of emerging materials and approaches in the development of smart thermally conductive fiber materials. Structural materials are classified based on their functions. Finally, they discuss the challenges and opportunities presented by smart thermally conductive fiber materials and present prospects for their future development.

## 3. Conclusions

Carbon-based multifunctional nanomaterials are expected to continue to play a pioneering role in materials science in the future and have great potential, particularly in the fields of electronics, energy storage, environmental protection, and biomedicine. Despite their enormous potential, current synthesis technologies face challenges such as high costs, difficulties in mass production, and performance instability, which require further technological breakthroughs. The future focus is on improving the manufacturing processes of carbon-based multifunctional nanomaterials to improve their quality, consistency, and controllability to meet the requirements of large-scale industrial production. Meanwhile, advances in functionalization techniques will provide these with materials improved competitiveness for certain applications. The performance of carbon-based multifunctional nanomaterials is also expected to be further improved through their integration with other functional materials, especially in the development of novel electronic devices, sensors, and efficient energy storage systems. In addition, with the increasing demand for environmentally friendly materials, the sustainability and environmental friendliness of carbon-based materials is also increasing.
